# Competency-based medical education and scholarship: Creating an active academic culture during residency

**DOI:** 10.1007/s40037-015-0218-4

**Published:** 2015-10-08

**Authors:** James A. Bourgeois, Ana Hategan, Amin Azzam

**Affiliations:** 1 Department of Psychiatry/Langley Porter Psychiatric Institute, Consultation-Liaison Service, University of California San Francisco Medical Center, San Francisco, USA; 2 Department of Psychiatry & Behavioural Neurosciences, Division of Geriatric Psychiatry, Michael G. DeGroote School of Medicine, Faculty of Health Sciences, McMaster University, St. Joseph’s Healthcare Hamilton, West 5th Campus, 100 West 5th Street, ON L8N 3K7 Hamilton, Canada; 3 Department of Psychiatry, University of California, San Francisco, USA; 4 School of Public Health, University of California, Berkeley, USA

**Keywords:** Competency, Medical education, Scholar role, Life-long learning

## Abstract

The competency-based medical education movement has been adopted in several medical education systems across the world. This has the potential to result in a more active involvement of residents in the educational process, inasmuch as scholarship is regarded as a major area of competency. Substantial scholarly activities are well within the reach of motivated residents, especially when faculty members provide sufficient mentoring. These academically empowered residents have the advantage of early experience in the areas of scholarly discovery, integration, application, and teaching. Herein, the authors review the importance of instituting the germinal stages of scholarly productivity in the creation of an active scholarly culture during residency. Clear and consistent institutional and departmental strategies to promote scholarly development during residency are highly encouraged.

Medical education systems are rooted in century-year-old traditions, including a ‘time based’ model of medical education (e.g., demonstrated time spent in residency programmes) [1]. Competency-based medical education (CBME) leads to a decreased emphasis on time-based learning by a greater emphasis on the accomplishment of developmental milestones, which could conceivably lead to shorter training times [2].

The CBME movement is ‘in full swing’ in medical education systems in many nations (e.g., United States, Australia, the Netherlands, Canada) [1, 3–5]. The attainment of ‘competence’ in a specific curricular area requires an integration of knowledge, skills, and behaviours in practice [2]. Since CBME promotes self-direction for the learning process, residents will have more freedom to decide *how* to learn and be more *actively* involved in the educational process, but they may have less freedom in deciding *what* to learn as competencies become categorized and standardized [2]. Therefore, CBME provides a clear description of intended outcomes but does not specify particular learning strategies or formats [2], at least at this current stage of CBME implementation. Faculty members at training programmes need to help residents go beyond simply graduating from the training programme. It is necessary to prepare residents to flourish over a career that includes continuing education and self-improvement, often under the rubric of ‘life-long learning’ [6]. A contemporary challenge therefore is to find ways to integrate research and other scholarly activities into the competency-based curricula.

The Accreditation Council for Graduate Medical Education (ACGME) in the United States emphasizes educational outcomes in the evaluation of residency programmes through this skill of life-long learning as one of the core accreditation competencies called ‘Practice-Based Learning and Improvement’ [7]. In Canada, the Draft CanMEDS 2015 Physician Competency Framework [6] has re-examined the seven core CanMEDS domains; the scholar role not only integrates life-long learning, but also critical appraisal, teaching, and research. The Competence by Design project of the Royal College in Canada is intended to implement an enhanced model for CBME starting with the residency training and moving into speciality practice [6]. This project will position Canadian medical education as the first in the world to integrate CBME across the full continuum of a physician’s career [6]. Therefore, CBME has the potential to provide a seamless linkage among all stages of life-long learning in medical practice. CBME involves implementing outcomes-driven curricula and education early in the training process and assessment to ensure residents and then speciality physicians possess the knowledge and abilities they need for every stage and role of their career [2]. How the centrally placed role of ‘medical expert’ interlinks with the other ‘intrinsic roles,’ a term describing the other CanMEDS roles (including the scholar role), in the CBME framework remains to be ascertained in detail.

Over the past two decades there has been a decline in faculty academic recruitment into more traditional research activities; multiple factors such as regulatory, institutional, funding, and personal barriers have been implicated [8, 9]. The Institute of Medicine has focused on the need for competency-based curricula that promote research training during residency as a key period of development of research interest and as a means to address the physician-scientist shortage [9].

The CBME model has the potential to add the competency of ‘scholarship’ as an important institutional expectation. This may be implemented in a broad way, herein the expectation of ‘scholarship’ is inclusive of traditional ‘research’ but also includes other critical areas of scholarship. Glassick [10] summarized Boyer’s four ‘meanings’ of scholarship: discovery, integration, application, and teaching. He also discussed the Carnegie Foundation for the Advancement of Teaching’s six standards of excellence in scholarship: clear goals, adequate preparation, appropriate methods, outstanding results, effective communication, and reflective critique as applied to the four ‘meanings’.

However, through the benefits of the CBME, the new training model may appear to provide a more personalized learning experience for residents, including implementing scholarly activities, than the traditional time-based approach [1]. In this view, Grady et al. [11] studied 30 medical schools in the United States in which the four Boyer elements (discovery, integration, application, and teaching) were emphasized. They developed a rubric to guide US residency review committees of the ACGME based on specific items exemplifying the four Boyer criteria as pertains to resident academic activity, as well as for assessing faculty members’ performance. Scholarship about educational initiatives can be a separately emphasized endeavour, and progress in this area can be an important departmental priority.

Promoting both scholarly activity (including but not solely limited to research training) during residency and the subsequent growth of academically oriented physicians has multiple implications. Academic currency is essential in an age of information explosion. One of the important aspects of academic life is publication in peer-reviewed journals and other media. Failure to publish can be a ‘rate limiting step’ in career development of academic physicians especially at certain institutions that persist in the ‘publish or perish’ philosophy (not necessarily explicitly stated as policy). There has been a trend towards broadening the content areas for scholarly endeavours beyond a previous emphasis primarily on foundational sciences, translation, and clinical interventions, to be inclusive of education activities (e.g., educational methods and curricular development) as well as other ‘scholarly substrates.’ With newly available methods for quantitative analysis and access to ‘big data’, areas of desirable research endeavours now also include quality assurance, the use of electronic medical records, population health, and systems of care themselves.

Establishing a pattern of academic productivity during residency can identify the strengths and innate talent of each resident, provide opportunities for those strengths to be fostered, and jump-start a successful academic career. The importance of instituting the germinal stages of scholarly productivity in the creation of an active scholarly culture during residency cannot be overestimated. The majority of residents will *not* participate in a structured academic or research track (models that involve a selection process and sequestered time for these activities separate from clinical service duties), yet they still need to master the skills of academic productivity, usually based on consolidation of their clinical experiences. With the rapid advances of digital technology in the academic publishing system, there is almost no area of scholarly practice that remains impervious to change. A goal of one or more scholarly products (broadly defined in terms of topic area) before residency graduation might be a realistic expectation in the development of a robust academic culture. Such an early establishment of the habit of publication and other forms of dissemination can facilitate ongoing productivity. In a Canadian national survey [12], successful completion of a research project during residency was found to be predictive of further participation in research projects after graduation. Graduates from programmes with formal research curricula have been more likely to report that their residency research project is a positive learning experience [12].

While resident scholarly activity can include a wide range of work products (e.g., peer-reviewed articles, letters to the editor, monographs, book chapters, poster presentations at conferences), increasing scholarly activity successfully requires certain vital components. Training programme features associated with successfully promoting resident academic productivity have included a system to engage faculty mentors, a formal research curriculum, a forum to present projects, technical assistance, dedicated research time, and funding support [13, 14]. A few key attributes that have been listed in the literature to potentiate resident success in research and scholarship are enumerated in Box 1 [9, 13, 15–20]. Training programme directors and department chairs/vice chairs must explicitly acknowledge that promoting and implementing an active research and scholarship culture during residency is resource-intensive. Schott et al. [21] have recently shown that residents’ dedicated research electives and tracks required significant departmental financial support. Moreover, residents who elected to spend dedicated months conducting research did complete significantly more scholarly projects than their peers but experienced fewer clinical cases, which may impact the accomplishment of other clinical competencies [21].

Historically, the educational culture at many residency training programmes has been driven by a primary focus on clinical care delivery. Limiting resident duty hours to regulatory maxima to improve patient safety and resident well-being, but also resident education, has been proven to be challenging [22, 23]. As such, ‘duty’ time devoted primarily to academic productivity may be sacrificed in the course of conforming with overall regulations on duty hours. However, a recent randomized trial of residents in Canadian ICUs did not support the purported advantages of shorter duty schedules [24]. Moreover, a systematic review and meta-analysis performed to evaluate the impact of resident duty hour limits on clinical and educational outcomes in surgery has shown that recent changes in resident duty hours have negative impacts on patient outcomes and also on performance on certification examinations [23]. Although there is a variation in training needs, diversity of practice patterns, and various competencies required among specialities, those authors advised that greater flexibility to accommodate resident training needs is required while further erosion of training time should be considered with great caution [23]; this is especially important to consider when ‘competency’ is ‘the holy grail’ of achievement.

Since the evidence around the necessity for protected time to engage in scholarly pursuits seems compelling (Box 1), there is a risk that CBME will shift us substantially away from being able to ‘carve out’ protected time for scholarly or research pursuits, unless the scholarly competency is itself independently prioritized. Zibrowski et al. [25] addressed the common theme of ‘not having the time’ for scholarly productivity in academic medical centres. As this is a thorny issue without a clear single solution, departments need to consider a number of local institutional support interventions to promote scholarship. Given the work-hour restrictions, this might suggest to some that residents have more time to *actually do scholarly* pursuits as an extracurricular approach during the off hours, as they are time-limited regarding their clinical work. As is oft true of faculty members themselves, significant time outside of the ‘duty day’ may inevitably be needed for working on academic products; i.e., there can never be ‘enough protected time’ solely during the duty day for optimized academic productivity. We will need to teach our residents to integrate their research/scholarly competencies alongside their clinical competencies in the view that we might not be able to completely ‘protect’ their scholarly time, modelling behavioural patterns of academically successful faculty members.

Box 1 Various key attributes shown to potentiate resident success in research & scholarship [9, 13, 15–20]

**Table:** 

Key components	Successful interventions
Mentoring	Providing support and encouragement, especially by clinical supervisors, and identifying research mentors are crucial. The most important factors for promoting resident research are
	• Availability of experienced research mentors (e.g., lack of local mentoring is one of the most often cited arguments against requiring all residents to participate in research [18])
	• Developing collaborative relationships of programmes without qualified faculty with programmes where expertise is available
	• Strong local department proponent for research;
	• Programme director support
Education	• Create a culture of inquiry that should begin early in residency when research interest is greatest [19]
	• Integrate research discussions into all educational forums
	• Resident physicians should be well versed in the principles of scholarship. This will enable producing scientific knowledge, critically evaluate the medical literature, and provide better quality care to patients
	• Offer forum to present projects; the venue is often a local research day, but present at any level of professional meeting as an opportunity to network, build confidence, and stimulate continuing engagement in research since many regional, national, and international conferences include spots for resident research to be presented
Experience	• The educational experience is the most relevant part of the project
	• Recommend research projects that do not require grant support
	• Simple study designs are preferable for educational purposes; studies requiring less time, less resources, and less money are more likely to be completed; the population should be one regularly encountered by the resident
	• Collaboration with other residents or faculty will lessen the workload, spread the educational experience, and increase the chances of completion
Time	• Dedicated time for faculty is crucial, so that faculty members involved in research act as role models and mentors to generate an atmosphere conducive to scholarship
	• Dedicated research time for residents has been deemed an indispensable factor in developing a productive resident research programme [20]
Support	• Financial considerations are one of the most frequently cited barriers to implementing a research curriculum [20]
	• Statistical expertise, administrative assistance, editorial assistance, and technical support are important for the success of early career investigators

For faculty mentors, the effort can result in increased numbers of their own publications, and lead to future collaborations, which enhances the faculty members’ own academic competitiveness. However, protected mentoring time for faculty to support residents remains elusive. Sambunjak et al. [26] completed a comprehensive review of mentoring in academic medicine, and found that the evidence supporting the prioritization of mentoring in medical education is not strong, despite a widespread belief in its general intrinsic value. Notably, only 50 % of medical students and 20 % of faculty had an identified mentor. Mentors were recommended to focus on mentees’ personal development, career choice, and research productivity. Mentorship should be widely available through educational periods and beyond, well into career development. Levy et al. [27] summarized a major initiative in an academic medical centre department of Internal Medicine to simultaneously facilitate career development of faculty members and residents. An important element of this study was the specific reward and prioritization of mentorship of residents by faculty with a formal mentorship programme (including metrics for mentorship and rewards for successful mentoring). Participating faculty members were expected to make a 3-year commitment to the mentorship role. Subsequently, both ‘assigned’ and ‘unassigned’ mentoring increased.

Van Melle et al. [28] assessed the promotion policies of the 17 Canadian medical schools and found that only 9 specifically referenced educational scholarship in promotion actions, and that these 9 universities had a wide variation in how they assessed and rewarded educational scholarship. They suggested that more specific guidelines be developed for the structured assessment of educational scholarship. Simpson et al. [29] described in detail the development of an Educator’s Portfolio at Medical College of Wisconsin to centralize and quantify educational scholarship activities for faculty members. They discussed the various steps in adopting this including creating a guiding coalition, developing vision and strategy, generating short-term wins, and anchoring the novel approach in the local academic culture. LaMantia et al. [30] described the Council of Emergency Medicine Residency Directors (CORD) and their product, the Academy for Scholarship in Education in Emergency Medicine, to promote educational achievement in emergency medicine.

While producing of publications is one objective indication of scholarly activity, other areas need to be developed to assure the maintenance of life-long learning and other integrative skills. An integrated approach to scholarly activity, with an inclusive and broad list of topics for research inquiry, and integrating the lessons derived from these activities, need to be modelled by faculty members and then adopted by residents. In this vein, residents must know that there are multiple opportunities for engaging in academic scholarship. Finding forums for opportunities to network and stimulate continuing engagement of residents in research may even be facilitated by the use of technology of the digital era we live in. Although the concept of ‘learners as digital natives’ has recently been questioned [31], the current residents who are greatly skilled and comfortable accessing information digitally represent a significant proportion of the *digital natives* (a term coined by Prensky [32]), or *homo zappiens* [33], *Google generation* [34] and so forth, terms given to the generation that does not know a world without computers, mobile phones, and the Internet [31]. Therefore, by converting those from ‘digital consumers’ to ‘digital contributors,’ it may soon become a professional norm to be contributing to the dissemination of knowledge in the ‘digital space,’ be that in peer-reviewed journals or non-peer reviewed blogs. A balanced emphasis on the various aspects of scholarship needs to be integrated into a balanced and timely set of clinical problem-solving skills, both at the patient level and the broader system level.

In summary, in the new era of CBME, creating an active academic culture during residencies does matter. Effective strategies to promote both scholarly/research training during residency and the growth of academically oriented residents are valuable pathways to pursue. Residency training programmes are encouraged to make academic productivity a major curricular goal, with a stance of inclusion of multiple opportunities for residents to create academic products in both the traditional (e.g., journal articles) and the modern (e.g., digital publications and other non-traditional contributions) methods of dissemination of professional communications. Residents with such experiences may be better prepared to flourish in academic positions from the beginning of their faculty careers.

**Declaration of interest**

The authors report no conflicts of interests. The authors report that there is no source of funding.
